# Pulmonary Arterial Hypertension Due to NPR-C Mutation: A Novel Paradigm for Normal and Pathologic Remodeling?

**DOI:** 10.3390/ijms20123063

**Published:** 2019-06-22

**Authors:** Emmanuel Eroume-A Egom

**Affiliations:** St Martha’s Regional Hospital, Dalhousie University, Antigonish, B2G 2G7 NS, Canada; egomemmanuel@gmail.com

**Keywords:** Idiopathic Pulmonary Arterial Hypertension (IPAH), Natriuretic Peptide Clearance Receptor (NPR-C) signaling

## Abstract

Idiopathic Pulmonary Arterial Hypertension (IPAH) is a deadly and disabling disease characterized by severe vascular remodeling of small pulmonary vessels by fibroblasts, myofibroblasts and vascular smooth muscle cell proliferation. Recent studies suggest that the Natriuretic Peptide Clearance Receptor (NPR-C) signaling pathways may play a crucial role in the development of IPAH. Reduced expression or function of NPR-C signaling in pulmonary artery smooth muscle cells may contribute to the pulmonary vascular remodeling, which is characteristic of this disease. The likely mechanisms may involve an impaired interaction between NPR-C, specific growth factors and other signal transduction pathways including but not limited to Gqα/mitogen-activated protein kinase (MAPK)/PI3K and AKT signaling. The resulting failure of growth suppression in pulmonary artery smooth muscle cells provides critical clues to the cellular pathobiology of IPAH. The reciprocal regulation of NPR-C signaling in models of tissue remodeling may thus provide new insights to our understanding of IPAH.

## 1. Introduction

Pulmonary arterial hypertension (PAH) is a devastating disease, which if not interrupted, leads to progressive right-sided heart failure and death within 2 to 3 years after diagnosis [1,2,3]. Although schistosomiasis may be the most common cause of PAH worldwide, evidence suggests that over half of cases of PAH in regions of the world without endemic schistosomiasis are idiopathic (IPAH) [4,5,6]. Pathobiologically, IPAH is a proliferative vasculopathy, characterized by vasoconstriction, vascular muscle cell proliferation, fibrosis, and microthrombosis [2,3]. Histologic findings may include hyperplasia and hypertrophy of all three layers of the pulmonary vascular wall (intima, media, adventitia) as well as fibrosis and in situ thrombi of the small pulmonary arteries and arterioles (plexiform lesions) [2,7,8]. It is during the transition from normal to remodeled pulmonary vascular cells that occur critical pathophysiological processes including, but not limited to, changes in key signal transduction pathways, from which the nitric oxide (NO) signaling is believed to be one of, if not, the major contributor to its homeostasis [2,3,9,10]. Several clinical studies have thus focused on targeting the NO pathway in patients with IPAH; however, they all fall short as to re-establishment of structural as well as functional pulmonary vascular integrity, as a basis for handicap-free long-term survival [11,12]. These findings could have been anticipated as individuals with IPAH are well known to have endothelial dysfunction and therefore reduced, if not, loss of NO signaling [12]. Interestingly, evidence also suggests that in the presence of a reduced or loss of NO signal transduction, there may be an enhanced natriuretic peptide clearance receptor (NPR-C)-mediated vasorelaxant effect [12,13]. They may thus be synergistic as well as complementary cardioprotective actions for NPR-C signal transduction and NO-mediated pathway in the vasculature [12,13]. The inhibition of one signaling pathway may therefore be compensated for by the upregulation of the other [12,13]. These striking observations raise the question of whether NPR-C signal transduction pathway may play a crucial role in IPAH pathobiology.

## 2. Normal NPR-C Signaling

**Figure 1:**
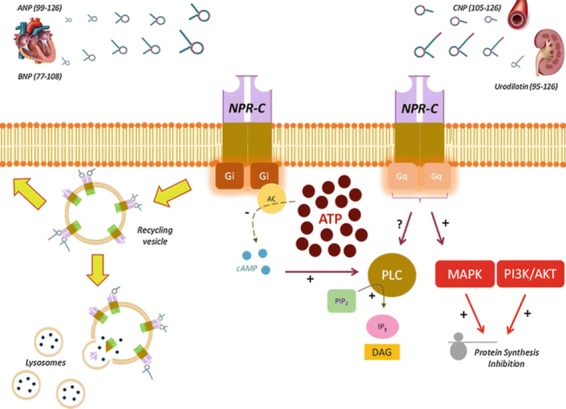
NPR-C signaling pathway. AC: adenylyl cyclase; ANP: Atrial Natriuretic Peptide; AKT: protein kinase B; ATP: adenosine triphosphate; BNP: Brain Natriuretic Peptide; cAMP: cyclic adenosine monophospate; CNP: C-type Natriuretic Peptide; DAG: diacylglycerol; Gi: G inhibitory protein; Gq: Gq protein; IP3: inositol triphosphate; MAPK: mitogen-activated protein kinase; PI3K: phosphatidylinositol 3 kinase; PIP2: phosphatidylinositol 4,5-bisphosphate; PLC: phospholipase C. +: stimulation; -: inhibition; ?: unknow. Reprint from [17] with permission from Springer.

Natriuretic peptides (NPs) constitute a family of at least four structurally related peptide hormones named Atrial Natriuretic Peptide (ANP), Brain Natriuretic Peptide (BNP), C-type Natriuretic Peptide (CNP), and Urodilatin (URO), which may regulate several biological processes including plasma volume and blood pressure control [10,14,15]. Most of the biological actions of NPs appear to be mediated via binding to three specific cell membranes receptors known as natriuretic peptide receptors-A, B, and C (NPR-A, NPR-B, and NPR-C) [10,14,15]. NPR-C is a disulfide-linked homodimer of a single transmembrane domain, an extracellular domain of ~440 amino acids; and a short 37 amino acid cytoplasmic domain with several inhibitory guanine nucleotide regulatory protein (Gi) activator peptide sequences that bind and activate the Gi-dependent signal transduction [16]. NPR-C has been found to lack the seven transmembrane domains of the typical G-protein-coupled receptors (GPCRs) and may thus be considered as an atypical GPCR [17]. NPR-C is widely distributed in several tissues and cells including but not limited to cardiac fibroblasts and myocytes, endothelial cells (EC) and vascular smooth muscles cells (VSMC) [16,17]. The binding affinity of the NPs for NPR-C is as follows: ANP > BNP > CNP [17,18]. Although originally classified as a clearance receptor with no signaling function, evidence suggests that NPR-C may be coupled to different intracellular signaling pathways including the adenylyl cyclase (AC)/cAMP signal transduction [16], the phospholipase C (PLC) signaling pathway, the nitric oxide (NO) pathway and Gqα/mitogen-activated protein kinase (MAPK)/PI3K and AKT pathways (As illustrated in Figure 1) [17,19,20,21].

Various studies have demonstrated that NPR-C can inhibit the AC/cAMP signal transduction and elicit physiological functions [22]. Natriuretic peptides such as ANP, CNP and BNP have been reported to inhibit AC and decrease cAMP levels in a variety of tissues and cells by interacting with NPR-C receptor [22,23]. In addition, activation of NPR-C signaling may decrease L-type calcium current in single sinuatrial node cells by AC inhibition and decreased cAMP levels [24]. Furthermore, the NPR-C’s agonist C-ANP_4–23_ may decrease the cAMP levels in VSMCs [23]. NPR-C has also been involved in the modulation of PLC signaling pathway. Phosphatidyl inositol turnover signaling is a major signal transduction pathway involved in intracellular calcium mobilization and protein kinase C (PKC) activation [16]. Evidence suggests that activation of NPR-C signaling may stimulate PI turnover in cultured bovine aortic smooth muscle cells [20]. Murthy and colleagues demonstrated that small peptide fragments of cytoplasmic domain of NPR-C may stimulate PLC-β activity in guinea pig tenia coli smooth muscle cells [23]. In addition, the NPR-C’s agonist C-ANP_4–23_ may trigger inositol triphosphate (IP3) formation in A10 vascular smooth muscle cells [25]. Whether the activation of PI turnover by C-ANP_4–23_ is a secondary event mediated through the AC/cAMP system coupled to NPR-C or a primary event is still not well understood. There may definitely be a cross-talk between NPR-C-mediated AC/cAMP and PLC signaling pathways as the inhibition of AC and decreased levels of cAMP triggered by NPRC activation may contribute to stimulation of PI turnover [16,25]. The modulation of other NPR-C-induced signal transduction pathways have also been suggested. As discussed in detail later, evidence suggests that NPR-C signaling may inhibit the platelet-derived growth factor (PDGF) and endothelin-3-induced mitogen-activated protein kinase (MAPK) through the inhibition of upstream kinases including MAPK kinase [26]. NPs antiproliferative effects in cardiac fibroblasts may be a result of NPR-C activation [27]. In addition, NPs-induced NPR-C signal transduction has also been implicated in modulating endothelial and VSMC proliferation, endothelial permeability in coronary endothelial cells as well as L-type calcium influx [17]. These observations suggest that the NPR-C signaling pathway may play a critical role in cell proliferation via ihinhibition of MAPK signaling pathway [16]. Finally, evidence also suggests that the NPR-C’s agonist C-ANP_4–23_ as well as other vasoactive peptides may trigger the activation of constitutive nitric oxide synthase (NOS) smooth muscle cells [28].

## 3. Consequences of Npr3 Mutation and/or Alterations in NPR-C Signaling

### 3.1. Studies in Transgenic and Knockout Mice

Both heterozygous and homozygous knockout mice for *Npr3* exhibit alterations in cardiovascular system, and several evidences suggest that mutations in *Npr3* may lead to cardiovascular diseases [17]. Recent evidence indicates a critical role of NPR-C signaling in the pathobiology of PH. We recently described the cardiac structure and function of mice lacking NPR-C (NPR-C^-/-^) by echocardiography [1,10]. NPR-C^-/-^ mice exhibit important structural features including right atrial and ventricular enlargement, hypertrophy of the right ventricular free wall, tricuspid regurgitation as well as echocardiographic findings suggestive of right ventricular pressure overload manifested as abnormal motion of the interventricular septum, which are all findings typically seen in humans with PAH [1]. Moreover, Doppler Echocardiography assessment demonstrated a significantly higher right ventricular systolic pressure compared with wild-type littermates [1]. These novel findings were also confirmed by resting right heart catheterization [1]. The above results suggest that NPR-C signal transduction may play a crucial role in the patho-biology of PAH.

### 3.2. NPR-C signaling in Hypoxia-Induced Pulmonary Hypertension

Group 3 Pulmonary hypertension (PH) includes PH due to lung diseases or hypoxia. Among the etiologies of group 3 PH, the strongest evidence favors hypoxic pulmonary vasoconstriction with remodeling of the pulmonary vascular bed. The pulmonary vascular remodeling is characterized by the proliferation, hypertrophy and extension of smooth muscle cells to previously unmuscularized pulmonary arterioles [29]. The mechanisms underlying hypoxia-induced PH and remodeling are poorly understood. One candidate that may play a critical role in the pathobiology of the condition is NPR-C signaling pathway. Evidence suggests NPR-C gene expression is selectively downregulated in the setting of hypoxia, which in turn may, at least in part, contribute to the development of hypoxic PH. Li and colleagues demonstrated that steady-state mRNA levels of NPR-C may be decreased to 20–30% of air control levels in lungs of hypoxia-adapted experimental models [30]. In addition, nuclear-runoff analysis revealed a huge and significant decreased transcription of the NPR-C gene in lung of hypoxia-exposed experimental models compared with air control, suggesting that the hypoxia-induced reduction of NPR-C steady-state mRNA levels is due to the downregulated gene transcription. As NPR-C in the lungs is located in pulmonary vascular smooth muscle cells (VSMCs), Sun and colleagues used pulmonary arterial smooth muscle cells (PASMCs) cultured in vitro to investigate mechanisms underlying the downregulation of NPR-C gene expression in lung of hypoxia-adapted experimental models [31]. The authors found that the diminished expression of NPR-C mRNA observed under hypoxic conditions in lung might be mediated through the tyrosine kinase receptor-associated fibroblast growth factor (FGF) and platelet-derived growth factor (PDGF), the gene expression of which is enhanced in lung of hypoxia-adapted animals [31]. Various FGFs and PDGFs are expressed in lung and may play critical roles in diverse aspects of pulmonary vascular remodeling, including but not limited to lung epithelial cells, VSMCs, and myofibroblast proliferation, differentiation, and angiogenesis, as well as adaptation to environmental hypoxia [31,32]. Evidence suggests that these growth factors may, at least in part, contribute to hyperproliferation of PASMCs and muscularization of pulmonary vasculature in hypoxia-induced PH [33,34,35,36]. The important relationship between the overexpression of these hypoxia-responsive growth factors and the downregulation of NPR-C has also been demonstrated in several studies performed in other experimental models [16,31]. Multiple signaling pathways may mediate the mitogenic activity of FGF and PDGF [31,37,38]. In fact, Sun and colleagues also demonstrated that the FGF- and PDGF-mediated downregulation of NPR-C gene expression is dependent on the activation of Ras-Raf-MEK-MAP kinase in PASMCs [31]. As activation of NPR-C signaling may have anti-proliferative effects, hypoxia-induced down regulation of NPR-C expression and associated impaired NPR-C pathways may lead to failure of the NPRC-related antiproliferative effects in the pulmonary vasculature, which may then ultimately lead to PAH [1].

### 3.3. Studies implicating NPR-C Signaling in Other Cardiovascular Diseases

Several evidence also suggests a potential role of NPR-C signaling pathways in the pathophysiology of other cardiovascular diseases [17,18]. Alterations in NPR-C signaling have been found in various experimental hypertensive models [17,27]. Consistently, a study in 200,000 European descents demonstrated an association between rs1173771 polymorphism in NPR-C with hypertension [39]. NPR-C signal transduction may also play an important role in the pathogenesis of atherosclerosis [17]. Recently, several novel single nucleotide polymorphisms (SNPs) of NPR-C were identified to be associated with atherosclerotic cardiovascular disease (ASCVD) in Han Chinese population [40]. Interestingly, multivariate logistical regression analysis demonstrated that the association between these SNPs and ASCVD remained significant even after adjustment for all the conventional risk factors of ASCVD including hypertension [40]. Although the underlying molecular mechanisms of the association between NPR-C gene polymorphism and ASCVD are unclear, some studies have shown that percutaneous coronary intervention-induced injury to neointima may lead to an increase in NPR-C expression in neointimal smooth muscle cells [17]. In addition, NPR-C is over-expressed near the luminal surface of atherosclerotic plaques and in VSMC [17]. After acute myocardial infarction, NPR-C expression may be up-regulated in infarcted and non-infarcted areas of the left ventricular wall [17]. NPR-C expression appears also upregulated in patients with heart failure [17,27]. Fox and colleagues identified four Npr3 SNPs that may be associated to left ventricular dysfunction, further suggesting an established effects of NPR-C variant on ASCVD [41].

## 4. NPR-C Signaling as a Therapeutic Target in Tissue Remodeling

Excessive vascular smooth muscle cell (VSMC) proliferation contributes to tissue remodeling that occurs in several vasculopathies including but not limited to PH [42,43]. Evidence suggests that activation of NPR-C signaling may attenuate vasoactive peptide-induced hyperproliferation of VSMC via MAP kinase and phosphatidylinositol 3-kinase (PI3K) pathways [44]. Consistently, Andalousi and colleagues have demonstrated that the NPR-C agonist, C-ANP_4–23_ may attenuate the enhanced proliferation of VSMC by decreasing the expression of cell cycle proteins [42]. The authors showed that the antiproliferative effect of C-ANP_4–23_ in VSMC was, again, mediated through the inhibition of MAP kinase/PI3-kinase/AKT signaling pathways [42]. Cahill and colleagues demonstrated that NPR-C may inhibit aortic smooth muscle cell mitogenesis and proliferation via inhibition of thymidine kinase activity [23]. In addition, NPR-C may mediate, at least partially, the anti-proliferative actions of BNP in human cardiac fibroblasts [45]. Sangaralingham and colleagues also demonstrated that NPR-C may mediate the antifibrotic and antiproliferative peptide CNP in cultured adult human cardiac fibroblasts [46]. Furthermore, we recently showed that mice lacking NPR-C exhibit enhanced collagen expression and deposition in the atria [47,48], whereas selective NPR-C activation may prevent pathological collagen deposition [49]. The activation of NPR-C signaling may inhibit the hypoxia-induced vascular endothelial cell growth factor (VEGF) transcription and protein production, suggesting that this receptor may have both direct [50], and indirect effects as antiproliferation factors for endothelial cells (EC), the latter potentially mediated via modulating VEGF synthesis [51]. Pedram and colleagues also demonstrated that NPR-C activation may inhibit the vasoactive peptides endothelin (ET)-stimulated secretion of VEGF and the subsequent EC proliferation and invasion [51]. These observations further support the potential therapeutic functions of NPR-C in vascular remodeling and angiogenesis [51]. Overall, the ability of the NPR-C signaling to impede cardiac or vascular remodeling may limit the cellular response to various chronic pulmonary vascular insults, making this pathway an attractive therapeutic target to prevent or reverse PH.

## 5. Conclusions

For several decades, NPR-C has been considered a NP clearance receptor responsible for receptor-mediated NPs degradation [17]. More recent data showing evidence of NPR-C multiple actions on different cells, organs and systems; and the existence of a specific intracellular signaling pathway have overcome this view. The role of NPR-C signaling in pulmonary diseases remains at an early stage. Although clearly of direct and critical relevance to PH, NPR-C signaling pathway is very likely to contribute to other pulmonary pathologies characterized by tissue remodeling, such as pulmonary fibrosis and chronic obstructive pulmonary disease. As activation of NPR-C signaling may have anti-proliferative effects, any alterations in this pathway may thus lead to failure of its antiproliferative effect within the pulmonary vasculature, which in turn may ultimately lead to pulmonary vascular remodeling. Large-scale and carefully designed genetic studies are needed to investigate alterations of NPR-C function and structure as well as the existence of gene polymorphisms in patients with PH, particularly patients with FPAH. Further studies are also required to completely elucidate the pathobiological role played by NPR-C signaling and to better understand how NPR-C alterations at gene and protein levels could contribute to tissue remodeling, particularly pulmonary vascular remodeling.
